# The innervation of the male copulatory organ of spiders (Araneae) – a comparative analysis

**DOI:** 10.1186/s12983-019-0337-6

**Published:** 2019-10-24

**Authors:** Tim M. Dederichs, Carsten H. G. Müller, Lenka Sentenská, Elisabeth Lipke, Gabriele Uhl, Peter Michalik

**Affiliations:** 1 Department of General and Systematic Zoology, Zoological Institute and Museum, University of Greifswald, Loitzer Straße 26, 17489 Greifswald, Germany; 2 Department of Botany and Zoology, Masaryk University, Kotlářská 2, 61137 Brno, Czech Republic; 3 German Air Force Center of Aerospace Medicine, Straße der Luftwaffe 322, 82256 Fürstenfeldbruck, Germany

**Keywords:** Copulation, Intromittent organ, Sexual selection, Bulb nerve, Sensory organ, Pedipalp, Palpal organ, Copulatory mechanism, Spiders

## Abstract

**Background:**

Nervous tissue is an inherent component of the many specialized genital structures for transferring sperm directly into the female’s body. However, the male copulatory organ of spiders was considered a puzzling exception. Based on the recent discovery of nervous tissue in the pedipalps of two distantly related spider species, we investigated representatives of all major groups across the spider tree of life for the presence of palpal nerves. We used a correlative approach that combined histology, micro-computed tomography and electron microscopy.

**Results:**

We show that the copulatory organ is innervated in all species investigated. There is a sensory organ at the base of the sperm transferring sclerite in several taxa and nervous tissue occurs close to the glandular tissue of the spermophor, where sperm are stored before transfer.

**Conclusions:**

The innervation of the copulatory organ by the bulb nerve and associated efferent fibers is part of the ground pattern of spiders. Our findings pave the way for unraveling the sensory interaction of genitalia during mating and for the still enigmatic mode of uptake and release of sperm from the male copulatory organ.

## Background

Animals with internal fertilization have evolved highly specialized and diverse genital structures for transferring sperm into the females´ body [1, 2]. These copulatory organs originate from different body parts, some being antecedent to the reproductive system such as penes and others being derived from fins, arms, legs or other body appendices [3–6]. They are everted or unfolded using muscles, hydraulics or both [7]. The supply of nerves is regarded as an inherent property of copulatory organs [1, 8, 9]. For example, in vertebrates, nerves play a pivotal role in the regulation of muscle contraction, exocrine secretion and blood flow e.g. [10]. The male copulatory organ of spiders, however, was considered a puzzling exception since no muscles, nerves and sense organs had been found in it [11–15].

In spiders, the paired male copulatory organs are situated on the pedipalps, which are paired body appendages anterior to the four pairs of walking legs. The use of pedipalps as intromittent organs in males is a synapomorphy for the Araneae [16]. At the tip of the male pedipalp, the so-called palpal organ (syns. ‘genital bulb’, ‘bulbus’) arises from the cymbium. The palpal organ can range from a simple tear-shaped structure to a complex set of sclerites and membranes. However, the structure of the palpal organ usually does not vary among conspecifics [17, 18]. The palpal organ contains the spermophor, an interim sperm storage site. How the sperm is taken up into the spermophor and released again during mating is one of the mysteries of spider reproductive biology [15, 18]. Before copulation, the palpal organ is inflated by hydraulic pressure, which causes complex shifts of the sclerites [14]. Most of these sclerites function as locking or bracing devices that interact with genital structures of the female [19, 20–23]. Once preliminary coupling is achieved, the embolus (often the only intromitting sclerite of the palpal organ) is maneuvered into the copulatory ducts of the female genitalia, which lead to the spermathecae where sperm are stored until egg laying.

The lack of innervation and muscles was explained by the notion that the palpal organ develops during ontogeny from the same epidermal cells that make up the tarsal claw, a structure devoid of nervous and muscular tissue [17]. It was accepted knowledge that the lack of nerves in the male copulatory organ was largely responsible for some of the peculiar features of spider mating behaviour such as a high occurrence of “flubs”, which were considered failed intromission attempts [17, 24]. The supposed lack of nerves also helped to explain the widespread existence of preliminary locking devices mentioned above [see ref. in 15]. Most recently, however, Quade et al. [25] report in a study on the development of the palpal organ that the “bulb primordium” forms beneath the base of the subadult claw. Although not explicitly addressed, this discovery questions the proposed insensitivity of the spider palpal organ.

Indeed, in the last few years, nervous tissue and a putative proprioreceptive embolic area were found in the palpal organ of the Tasmanian cave spider *Hickmania troglodytes* (Austrochilidae) [26], and neurons and a sensory organ were found in the palpal organ of the running crab spider *Philodromus cespitum* (Philodromidae) [27]. These studies show that a nerve enters the palpal organ from the cymbium and is connected with several clusters of neurons inside the palpal organ. The basis of the embolus of both species is innervated and *P. cespitum* possess an internalized sensory organ in this region. Furthermore, nervous tissue was found close to epidermal exocrine glands that discharge secretion into the spermophor.

**Fig. 1:**
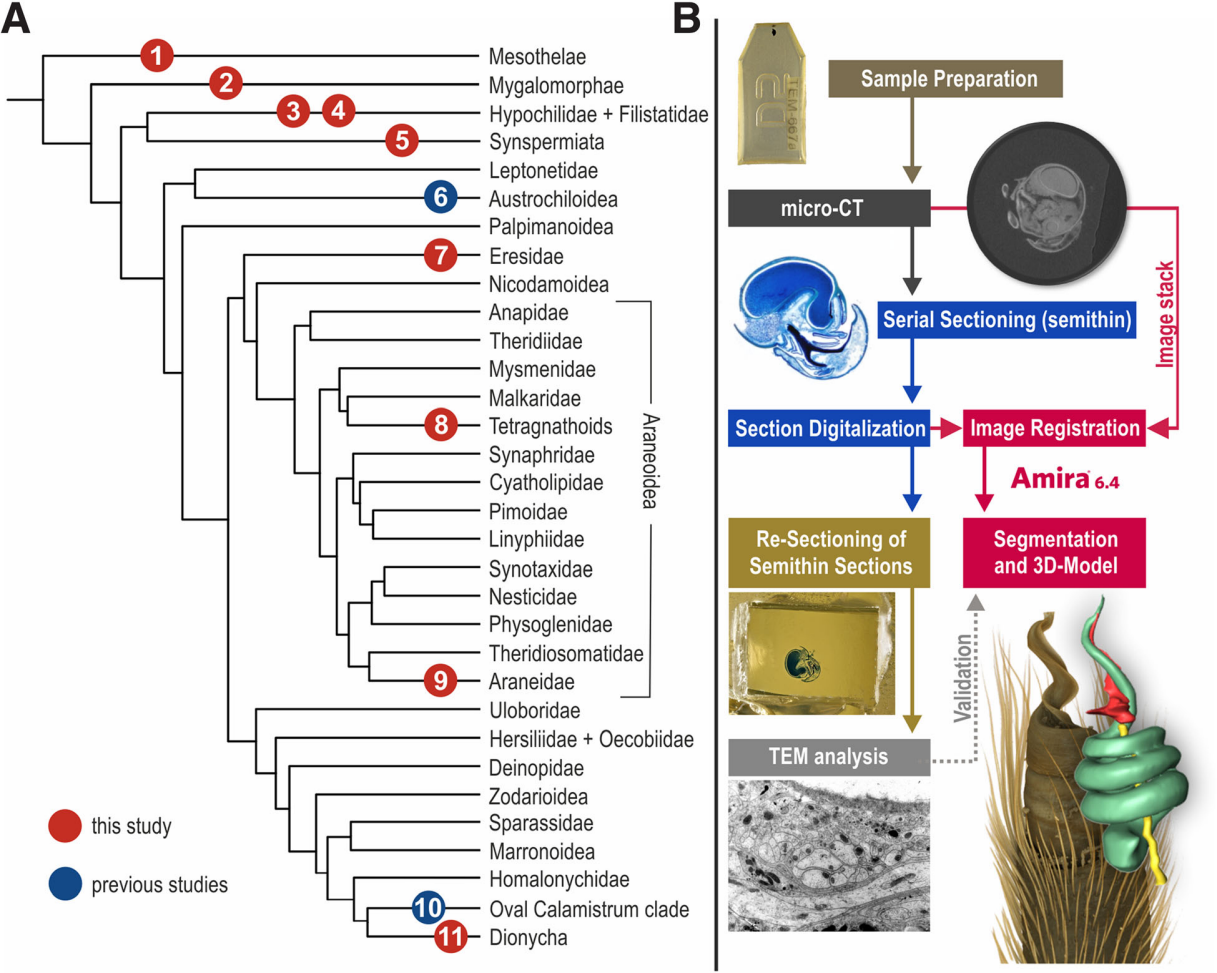
(**a**) Selected taxa for the present study (red) and previous studies (blue) on the innervation of the male copulatory organ of spiders. . 1: *Liphistius* sp.; 2: *Davus fasciatus*; 3: *Hypochilus pococki*; 4: *Kukulcania hibernalis*; 5: *Loxosceles rufescens*; 6: *Hickmania troglodytes* [26]; 7: *Stegodyphus dumicola*; 8: *Tetragnatha extensa*; 9: *Larinia jeskovi*; 10: *Philodromus cespitum* [27]; 11: *Marpissa muscosa*. (**b**) Flowchart of the correlative imaging approach used in this study

Following these findings, we investigated the organization of the palpal organ across the spider tree of life using a multimodal and correlative imaging approach combining histology, micro-CT and TEM (Fig. 1). This allowed us to test the hypothesis that the innervation of the palpal organ is part of the ground pattern of spiders. Novel comparative data also delivered insights on the diversity of innervation patterns in male spider copulatory organs.

## Results

We found nervous tissue in the palpal organs of all investigated taxa. The palpal organ is innervated by a branch of the pedipalp nerve, which enters from the cymbium through either a stalk-like connection between palpal organ and cymbium (*Liphistius, Davus, Hypochilus, Kuculkania, Loxosceles*) or the prominent basal haematodocha (*Stegodyphus, Larinia, Tetragnatha, Marpissa*). The entire course of the bulb nerve could not always be reconstructed due to fixation issues in some taxa (i.e., *Liphistius* and *Davus*). In all taxa investigated, the nerve appears to be associated and sometimes connected with one or several cell clusters. Glia cells and their ramifying projections surround and traverse the nerve and the neurite bundles merged therein along the way. In all araneomorph taxa, the neurite bundles run parallel to a small haemolymph vessel. Furthermore, each palpal organ contains up to three glands that are always connected to the spermophor. The spermophor appears to possess pores in some areas. We found that the clusters of neuronal somata are often situated very close to these glandular epithelia, but we cannot provide evidence for functional connections between neurites and glands. In line with previous investigations, we did not find muscles in the palpal organ of all taxa investigated.

### Mesothelae: Liphistiidae: *Liphistius* sp.

**Fig. 2:**
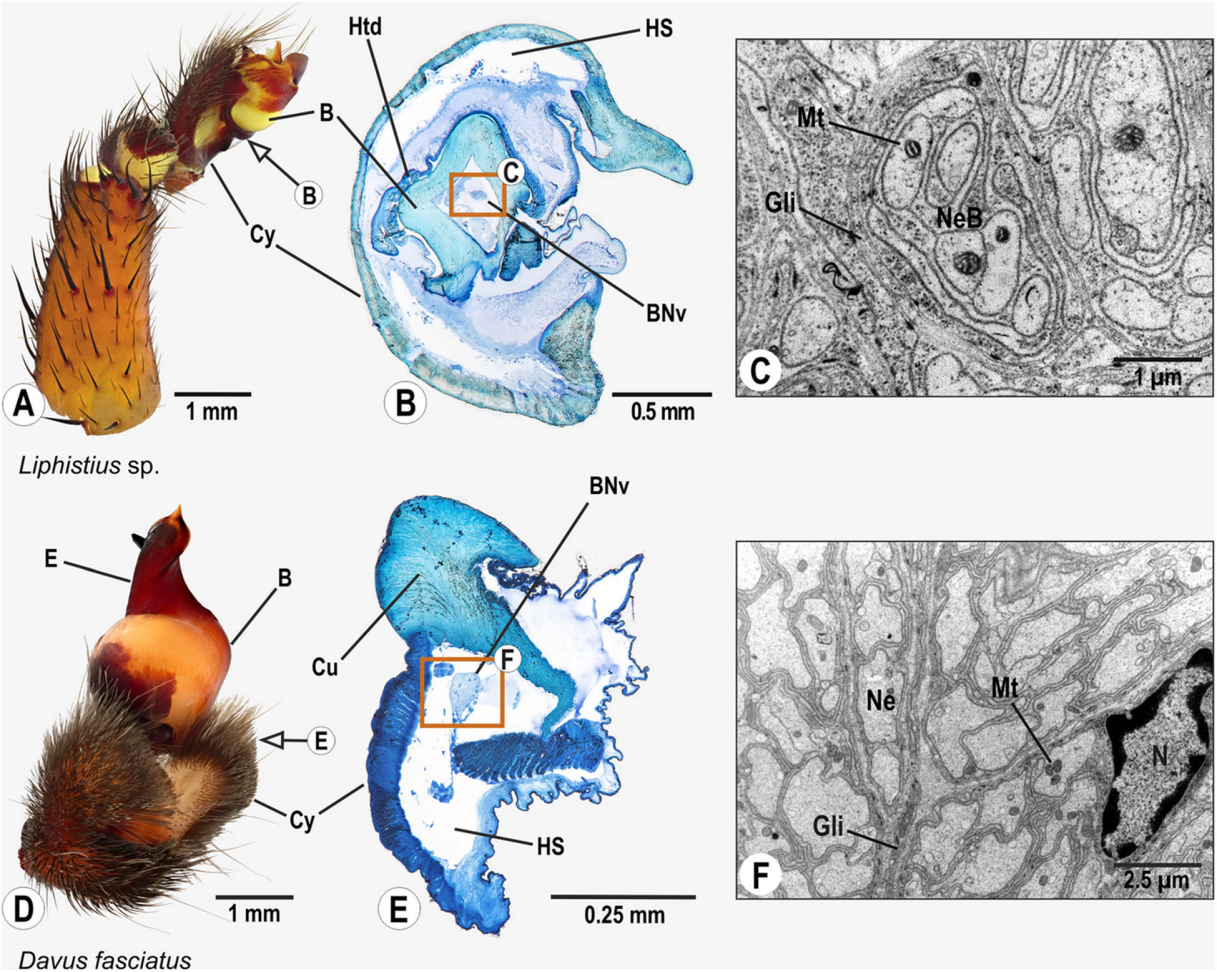
Palpal organs of *Liphistius* sp., and *Davus fasciatus*; external morphology (**a**,**d**), histology (**b**,**e**), and ultrastructure as documented by TEM (**c**,**f**). Arrows indicate planes chosen for semithin sections (**b**,**e**). Boxes in B and E show areas in the palpal organ where profiles of the bulb nerve were found. TEM micrographs in C and F show ultrastructural details of the bulb nerve. Abbreviations: B Bulb, BNv Bulb nerve, Cu Cuticle, Cy Cymbium, E Embolus, Gli Glial cell processes, HS Haemolymph space, Htd Haematodocha, Mt Mitochondrium, Ne Neurite, NeB Neurite bundle, N Nucleus of glial cell

The palpal organ is compact and partly retractable into the cymbium (Figs. 2A, B). It is connected to the cymbium via a strongly sclerotized tube. A nerve, defined as bulb nerve herein, branches off the palpal nerve in the cymbium and enters the bulb through this tube and projects further distally (Figs. 2B, C). Since sufficient fixation of the large palp was difficult, the further pathway of the nerve could not be reconstructed.

### Mygalomorphae: Theraphosidae: *Davus fasciatus* O. Pickard-Cambridge, 1892

The cymbium is divided into two lobes, which are richly equipped with setae (Fig. 2D). The palpal organ is situated between the cymbial lobes and connected to the cymbium by a short, stalk-like tube. The palpal organ is compact, tear-shaped and strongly sclerotized with a stout embolus (Fig. 2D). The bulb nerve projects from the cymbium into the palpal organ (Figs. 2E, F.) As in *Liphistius* sp., tissue fixation was not sufficient for a full reconstruction of this nerve.

### Araneomorphae: Hypochilidae: *Hypochilus pococki* Platnick, 1987

**Fig. 3:**
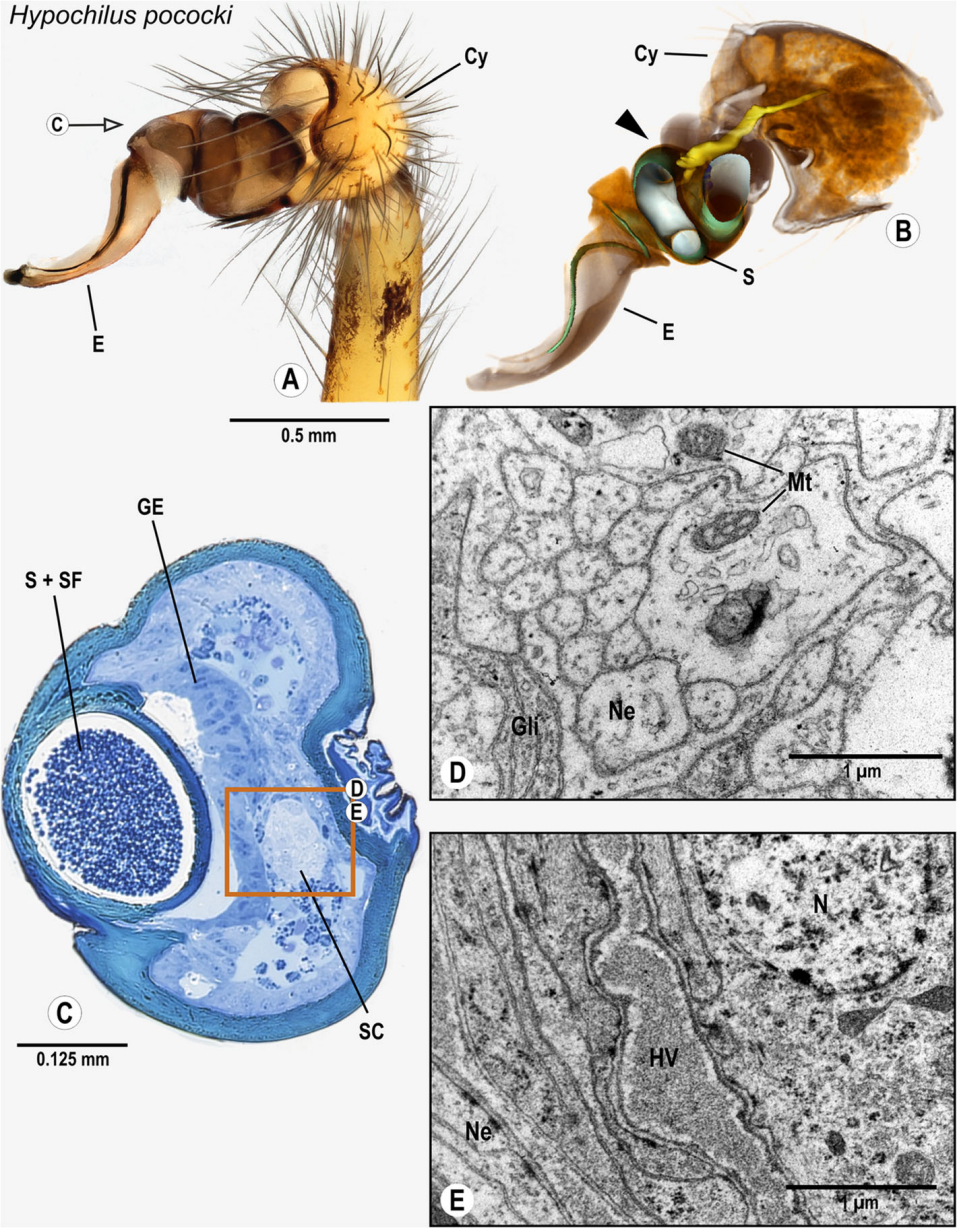
Palpal organ of *Hypochilus pococki*; external morphology (**a**), histology (**c**), ultrastructure documented by TEM (**d**, **e**), and 3D reconstruction of the spermophor (green) and nervous tissue (yellow) as based on segmentation of histological image stacks (**b**). The arrow indicates the plane chosen for semithin section (**a**). The arrowhead marks the terminals of the bulb nerve and associated neurons (**b**). The box marks the cluster of neuronal somata, and ultrastructural details of neurites bundles (**d**) associated with cluster of neuronal somata (**e**). Abbreviations: HV Haemolymph Vessel, Cy Cymbium, E Embolus, GE Glandular epithelium, Gli Glial cell processes, Mt Mitochondria, Ne Neurite, N Nucleus of a neuron, S Spermophor, SC Cluster of neuronal somata, SF Seminal fluid

The cymbium of *H. pococki* widens distally, forming a cup-like structure that encompasses the palpal organ (Fig. 3A). The palpal organ is relatively small and has a long and curved embolus. Inside the bulb the spermophor is convoluted and decreases in diameter from the base of the bulbus to the tip of the embolus (Fig. 3B). The bulb nerve projects from the cymbium into the palpal organ and is connected to a distinct cluster of neurons near the spermophor’s blind end, the so-called fundus (Figs. 3C–E). This cluster is situated directly beside a spermophor-associated gland. Laterally of the cluster, a small haemolymph vessel is present (Fig. 3E). The bulb nerve branches into small neurite bundles that run to the base of the embolus (Fig. 3B). Due to fixation problems, the exact pattern of these neurites could not be reconstructed in the most distal embolic part of the palpal organ.

### Araneomorphae: Filistatidae: *Kukulcania hibernalis* (Hentz, 1842)

**Fig. 4:**
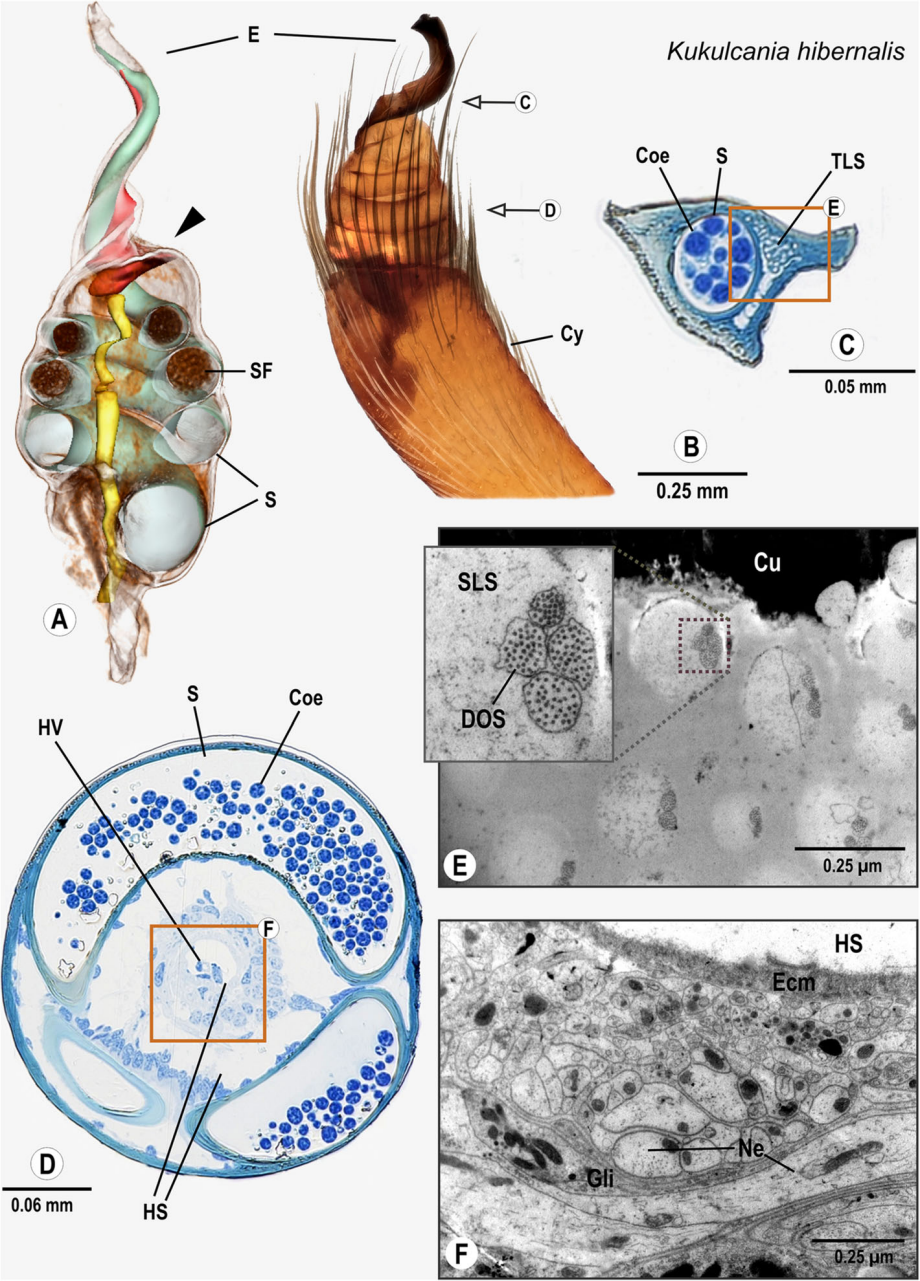
Palpal organ of *Kukulcania hibernalis*; external morphology (**b**), histology (**c**, **d**) ultrastructure as documented by TEM (**e**, **f**) and 3D reconstruction of the spermophor (green), nervous tissue (yellow) and internalized sensilla/“tube-like-structures” (red) as based on segmentation of histological image stacks (**a**). Arrows indicate the planes chosen for semithin sectioning (**b**) and the arrowhead marks the terminals of the bulb nerve (**a**). Box in C marks the sector with internalized aggregated sensilla magnified in (**e**), whereas box in (**d**) frames the region around the bulb nerve magnified partly in (**f**). Inset in (**e**) highlights four dendritic outer segments assembled in an internalized sensillum. Abbreviations: Coe Coenospermium, Cy Cymbium, DOS Dendritic outer segments, Ecm Extracellular Matrix, E Embolus, Gli Glial cell processes, HS Haemolymph space, HV Haemolymph vessel, Ne Neurite, S Spermophor, SF Seminal fluid, SLS Sensillum lymph space, TLS aggregated tube-like structures

The long and slender cymbium has an indentation at the distal end, in which the palpal organ is situated. Long setae around the margin of the indentation partly enclose the genital bulb. The palpal organ is tear-shaped with a spiral, broad embolus (Fig. 4B). At the stalk-like base of the palpal organ, the bulb nerve enters and projects through the bulbus. The bulb nerve runs through the centre of the bulbus accompanied by a small haemolymph vessel (Fig. 4D). The surrounding spermophor winds multiple times within the bulbus before projecting into the embolus (Fig. 4A). Between the third and fourth coil of the spermophor, the bulb nerve winds around a circular haemolymph space (Figs. 4A, D). In this region, it consists of several aggregated neurite bundles, which project in transversal and longitudinal direction (Fig. 4F). The neurite bundles are tightly encompassed and separated from each other by multiple sheaths of glial cell processes. The bulb nerve is separated from the haemolymph space by an extracellular matrix (Fig. 4F) and continues as far as to the base of the embolus. At the base of the embolus, tube-like structures can be found that run parallel to the spermophor towards the tip of the embolus (Fig. 4C). TEM analysis revealed that each of these tubular structures represent a thick dendritic sheath that encloses 2–4 dendritic outer segments embedded in an electron-lucent sensillum lymph space. This pattern is similar to the configuration described in tip-pore sensilla of the tarsal organ [27, 28] (Fig. 4E).

### Araneomorphae: Synspermiata: Sicariidae: *Loxosceles rufescens* (Dufour, 1820)

**Fig. 5:**
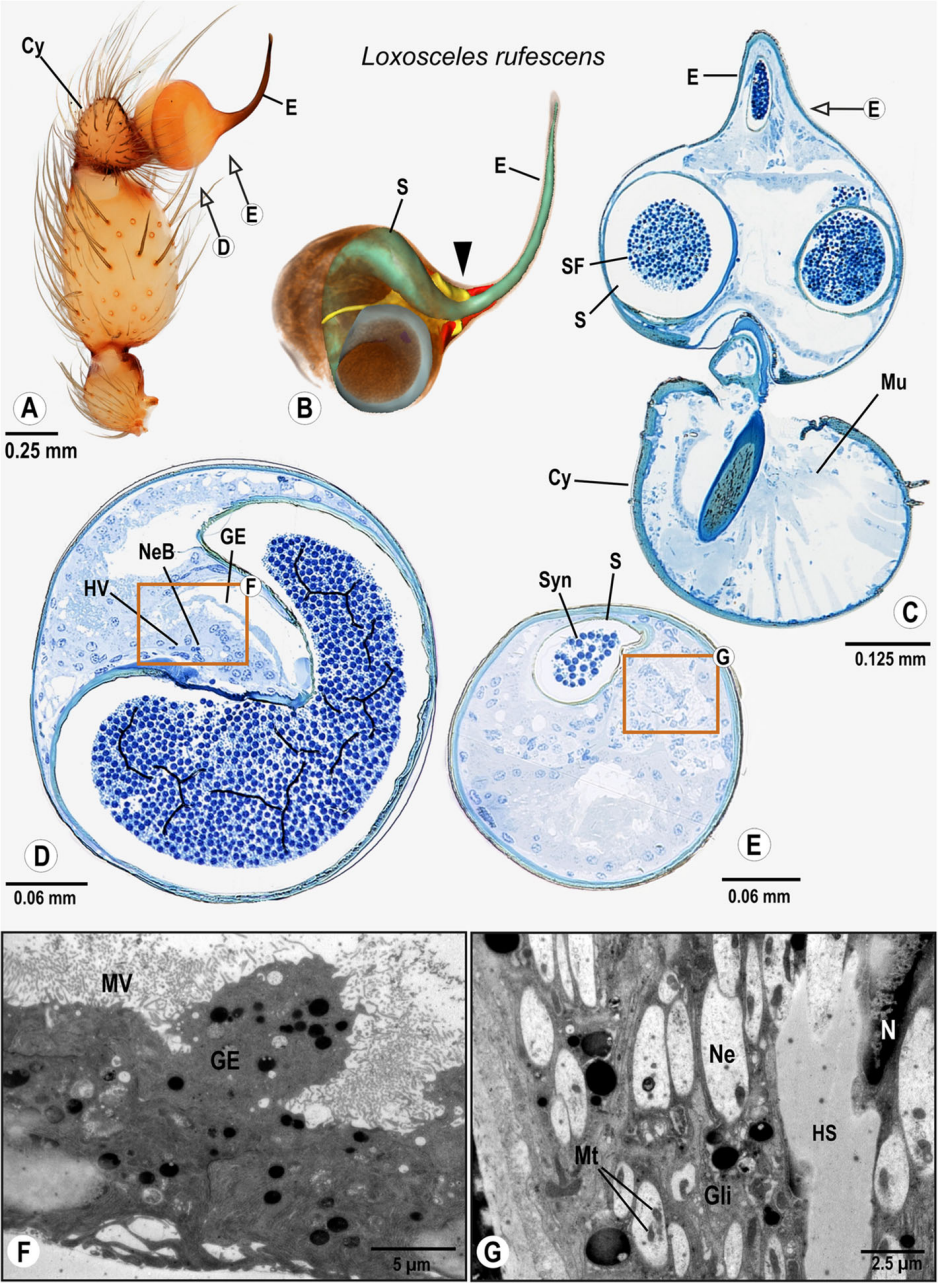
Palpal organ of *Loxosceles rufescens*; external morphology (**a**), histology (**c**–**e**) ultrastructure as documented by TEM (**f**, **g**), and 3D reconstruction of the spermophor (green), nervous tissue (yellow) and distinct cell clusters/“sensor epidermal tissue” (red) as based on segmentation of histological image stacks (**b**). Arrows indicate planes chosen for semithin sections (**a**) and the arrowhead marks the terminals of the bulb nerve (**b**). Box in (**d**) indicates the location of glandular tissue in the bulb, ultrastructural details are given in (F). Box in (**e**) marks the branches of the bulb nerve, highly magnified in G. Abbreviations: Cy Cymbium, E Embolus, GE Glandular epithelium, Gli Glial cell processes, HS Haemolymph space HV Haemolymph Vessel, Mu Muscle, Mt Mitochondria, MV Brush of microvilli, Ne Neurite, NeB Neurite Bundle, N Nucleus, S Spermophor, SF Seminal fluid, Syn Synspermium

The small cymbium carries a relatively large and simply structured palpal organ. The palpal organ is spherical and shows a slender, slightly bent embolus (Fig. 5A). The spermophor coils once before projecting into the embolus (Fig. 5B). From the cymbium, the bulb nerve projects into the palpal organ, and runs parallel to a haemolymph vessel before connecting to several clusters of neuronal somata near the base of the embolus (Figs. 5B, D, E). From these clusters, small neurite bundles project into the surrounding epidermal tissue between the spermophor and the cuticle of the palpal organ (Fig. 5G). This “sensory epidermal tissue” projects further into the embolus (Fig. 5B indicated in red; Figs. 5C, G). It is unclear whether it consists of glandular tissue.

### Araneomorphae: Eresidae: *Stegodyphus dumicola* Pocock, 1898

**Fig. 6:**
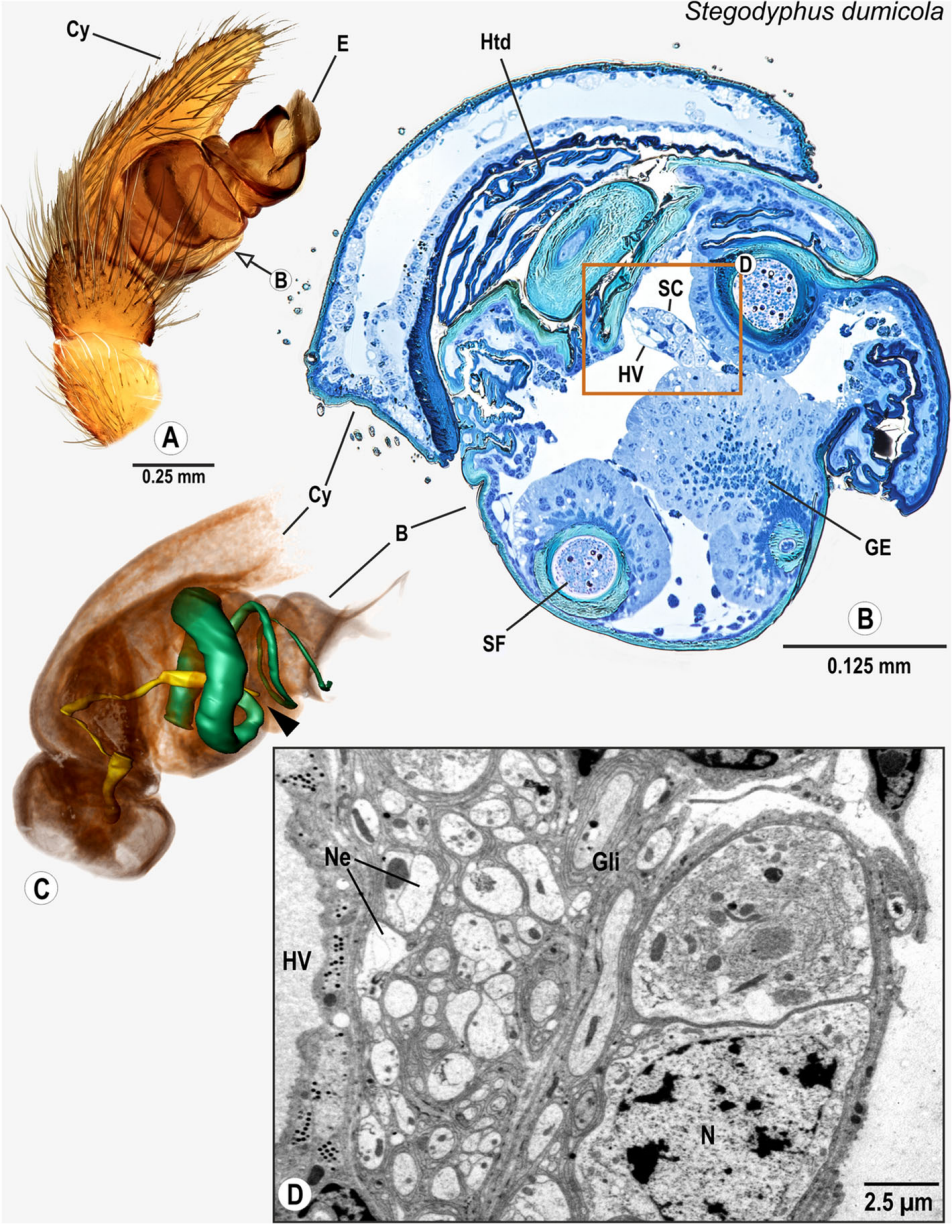
Palpal organ of *Stegodyphus dumicola*; external morphology (**a**), histology (**b**), ultrastructue as documented by TEM (**d**), and 3D reconstruction of the spermophor (green) and nervous tissue (yellow) as based on segmentation of histological image stacks (**c**). Arrow in (**a**) indicates the plane chosen for semi-thin cross section and arrowhead in (**c**) marks terminals of the bulb nerve. Box in (**b**) shows central position of bulb nerve in the palpal organ, surrounded by neuronal somata, ultrastructural details of boxed area are illustrated in (**d**). Abbreviations: B Bulbus, Cy Cymbium, E Embolus, GE Glandular epithelium, Gli Glial cell processes, HS Haemolymph space, Htd Haematodocha, HV Haemolymph Vessel, Mt Mitochondria, Ne Neurites, NeB Neurite Bundle, N Nucleus of a neuron, S Spermophor, SC Cluster of neuronal somata

The cymbium hosts the palpal organ in a spoon-like indentation. The palpal organ is compact and stout (Fig. 6A). The spermophor is a thin, winding tube (Fig. 6C). The bulb nerve enters the palpal organ and projects towards a large, spermophor-associated gland, where it connects with a cluster of neuronal somata (Figs. 6B, D). The somata cluster is adjacent to three distinct haemolymph vessels (Figs. 6B).

### Araneomorphae: Araneoidea: Araneidae: *Larinia jeskovi* Marusik, 1987

**Fig. 7:**
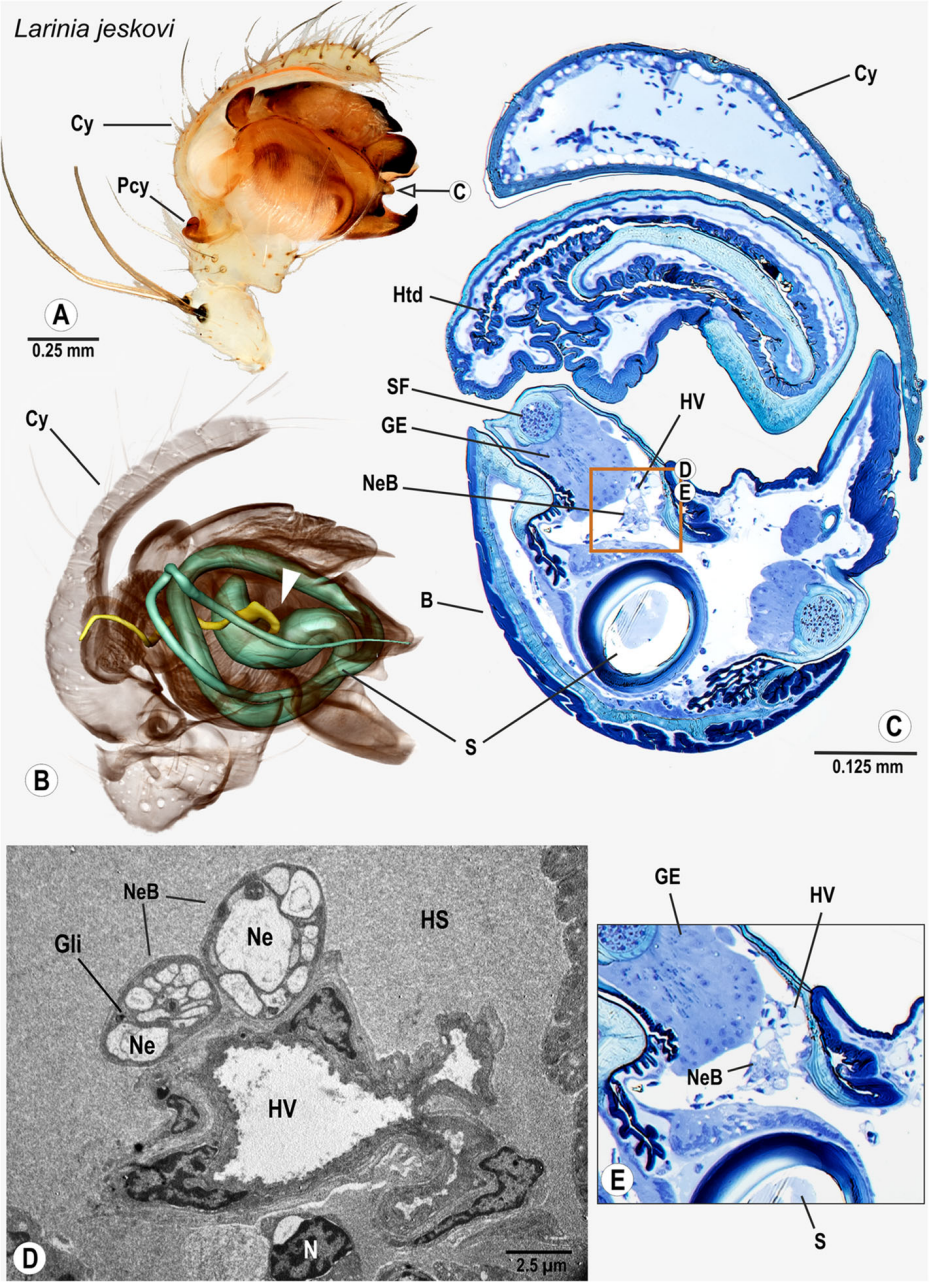
Palpal organ of *Larinia jeskovi*; external morphology (**a**), histology (**c**, **e**), ultrastructure as documented by TEM (**d**), and 3D reconstruction of the spermophor (green) and nervous tissue (yellow) as based on segmentation of histological image stacks (**b**). Arrow in (**a**) indicates plane chosen for semi-thin cross section (**c**). Arrowhead in (**b**) marks terminals of the bulb nerve. Box in (**c**) shows position and arrangement of neurite bundles branching off the bulb nerve, boxed sector is magnified in (**e**). Ultrastructural details of the same sector are given in (**d**), note the presence of two distinct neurite bundles adhering a haemolymph vessel. Abbreviations: B Bulbus, Cy Cymbium, GE Glandular epithelium, Gli Glial cell processes, HS Haemolymph space, Htd Haematodocha, HV Haemolymph Vessel, Ne Neurite, NeB Neurite Bundles, Pcy Paracymbium, SF Seminal fluid

The cymbium is thin, spoon-shaped and has a small paracymbium. The palpal organ is compact and bears several sclerites (Fig. 7A). The spermophor starts with an S-shape and performs two loops (Fig. 7B). The bulb nerve projects into the palpal organ through the basal haematodocha (Fig. 7B). In the bulb, nervous tissue can be found close to glandular tissue of the spermophor, forming a cluster of neurons (Figs. 7C, E). Throughout its course, the bulb nerve is associated with a small haemolymph vessel (Figs. 7C, D).

### Araneomorphae: Araneoidea: Tetragnathidae: *Tetragnatha extensa* (Linnaeus, 1758)

**Fig. 8:**
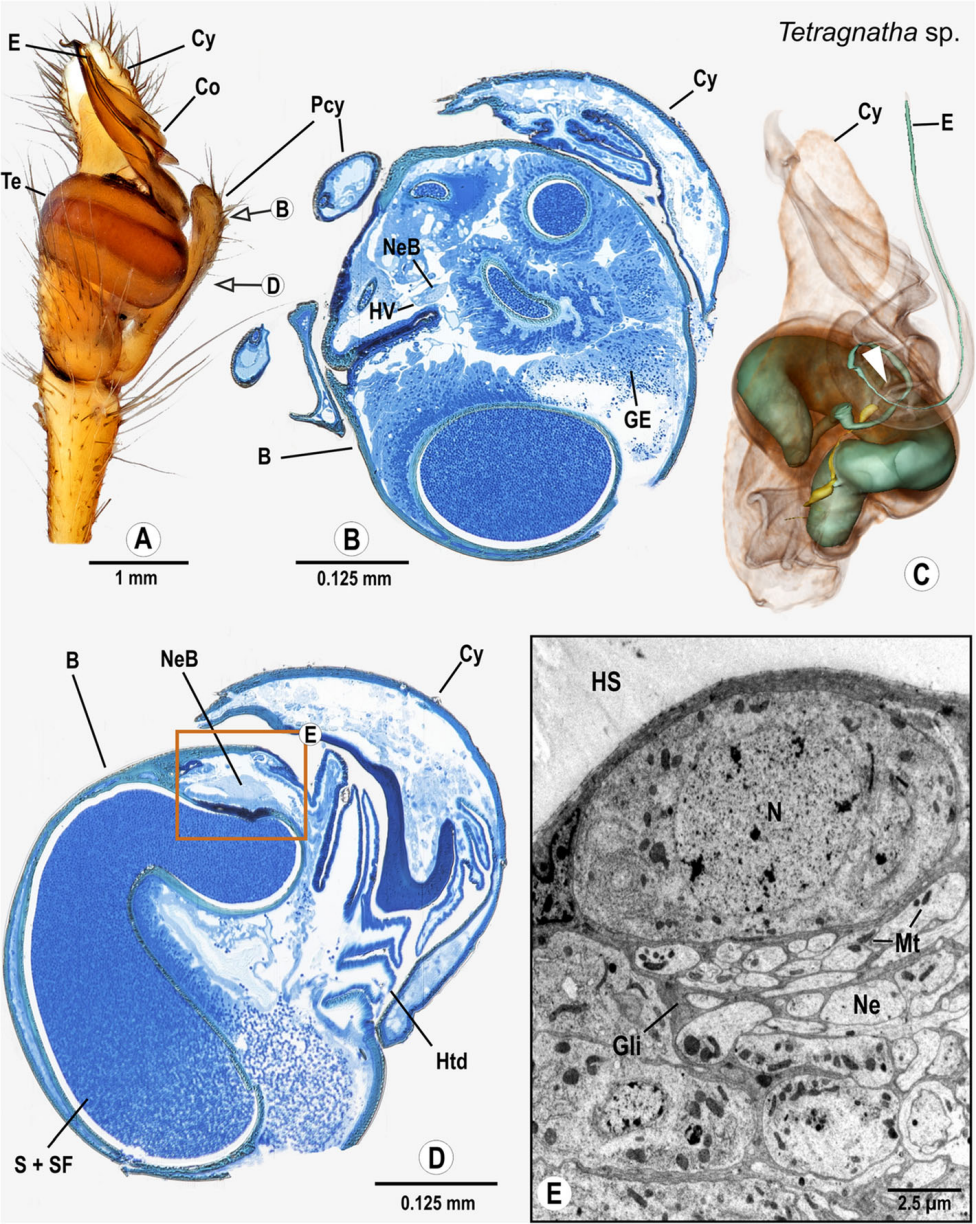
Palpal organ of *Tetragnatha montana*.; external morphology showing the general organization (**a**) as well as histology (**b**, **d**), ultrastructure as documented by TEM (**e**), and 3D reconstruction of the spermophor (green) and nervous tissue (yellow) of *Tetragnatha extensa* as based on segmentation of histological image stacks (**c**). Arrows in (**a**) indicate planes for semi-thin cross sections taken at distal end (**b**) and midlevel (**e**) of the bulb. Arrowhead marks terminals of bulb nerve (**c**). Note that the embolus in (**a**) rests in a ridge of the conductor and therefore differs from the one depicted in (**c**). Box shows a sector where neuronal somata and a neurite bundle branched off the bulb nerve are present, part of this sector is shown in (**e**) magnified to ultrastructural level. Abbreviations: B Bulbus, Co Conductor, Cy Cymbium, E Embolus, GE Glandular epithelium, Gli Glial cell processes, HS Haemolymph space, Htd Haematodocha, HV Haemolymph Vessel, Mt Mitochondria, N Nucleus, Ne Neurite, NeB Neurite Bundle, Pcy Paracymbium, S Spermophor, SF Seminal fluid, Te Tegulum

As in other *Tetragnatha* species (Fig. 8A), the pedipalps of *T. extensa* are characterized by a slender cymbium with a long paracymbium. The palpal organ consists of a spherical tegulum, a prominent and deeply ridged conductor as well as a long, thin and curved embolus. The spermophor is large in diameter and takes one turn before it narrows and enters the embolus (Figs. 8B, C). The bulb nerve enters the palpal organ from the cymbium through the basal haematodocha and is connected with two clusters of neuronal somata. The proximal cluster is situated near the basal haematodocha (Figs. 8D, E) in a distinct area between the bulbus-cuticle and the fundus of the spermophor. The other cluster is located more distally in the bulbus near a cuticular fold (Fig. 8B). Throughout its course, the bulb nerve is associated with a small haemolymph vessel.

### Araneomorphae: RTA clade: Salticidae: *Marpissa muscosa* (Clerck, 1757)

**Fig. 9:**
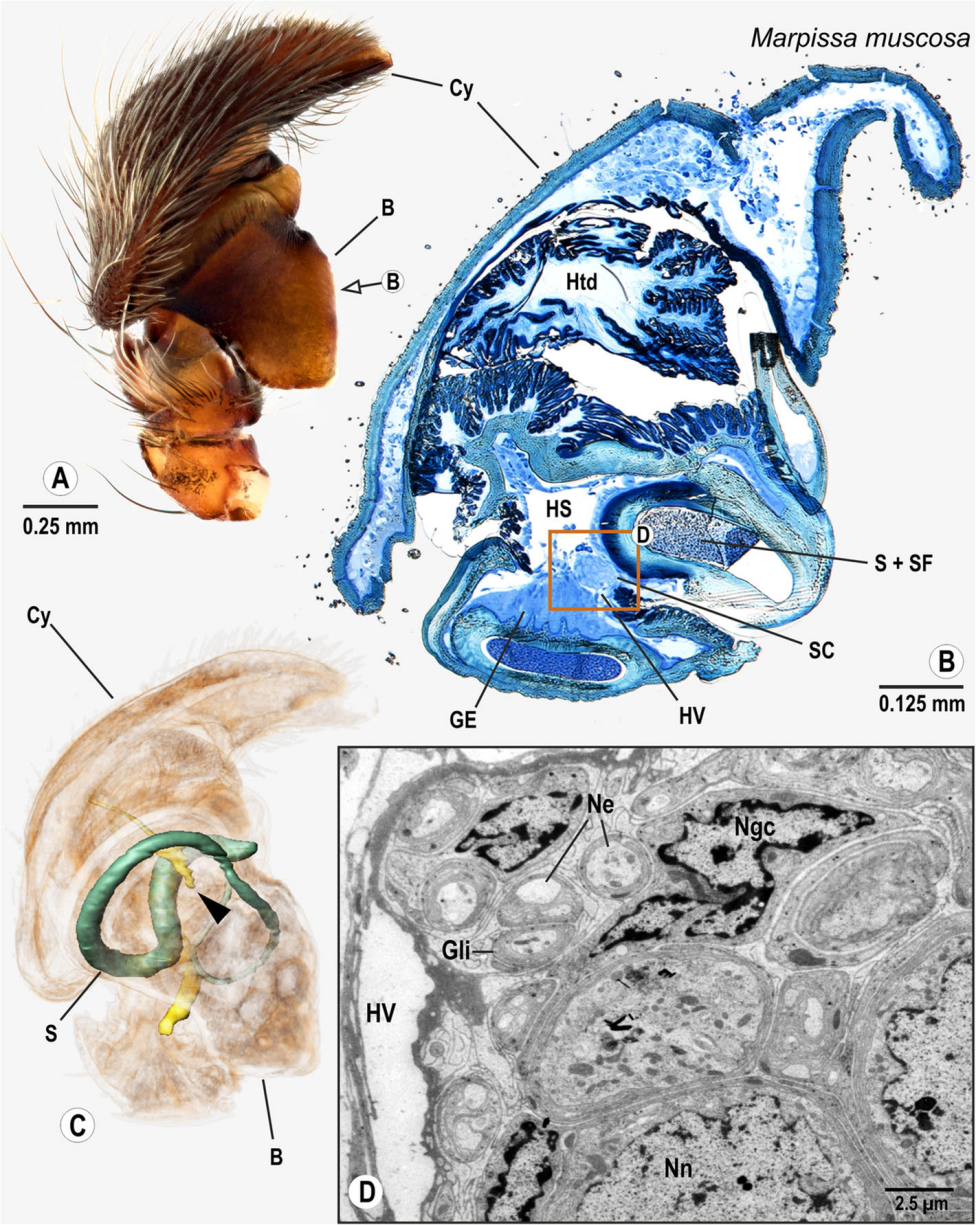
Palpal organ of *Marpissa muscosa*; external morphology (**a**), histology (**b**), ultrastructure as documented by TEM (**d**), and 3D reconstruction of the spermophor (green) and nervous tissue (yellow) as based on segmentation of histological image stacks (**c**). Arrow in (**a**) indicates plane for semi-thin cross section at midlevel of the bulb (**a**), arrowhead in (**c**) points at terminals of the bulb nerve. Box in (**b**) marks position of neuronal somata and closely adjoined neurite bundles branched off bulb nerve. (**d**) provides insights into ultrastructure of neuronal somata and neurite bundles. Abbreviations: B Bulbus, Cy Cymbium, GE Glandular epithelium, Gli Glial cell processes, HS Haemolymph space, Htd Haematodocha, HV Haemolymph Vessel, Mt Mitochondria, Ngc Nucleus of glial cell, Nn Nucleus of neuron, Ne Neurite, S Spermophor, SC Cluster of neuronal somata, SF Seminal fluid

The cymbium of *M. muscosa* is broad and hosts a strongly sclerotized palpal organ that extends backwards (Fig. 9A). The spermophor winds once inside the palpal organ (Fig. 9C). The bulb nerve enters the palpal organ after traversing the cymbium and the basal haematodocha. On its path through the palpal organ, the bulb nerve adjoins a small haemolymph vessel (Fig. 9D) and is connected to a single cluster of neuronal somata, which is situated close to the glandular tissue of the spermophor (Fig. 9B).

## Discussion

Our study reveals that there is a bulb nerve branching off the palpal nerve at the transition to the bulbus; this nerve projects into the palpal organ of all investigated taxa across the spider tree of life. Thus, our findings confirm our hypothesis that the innervation of the male palpal organ is part of the ground pattern of spiders. Moreover, the internal organization of the palpal organ, which has several spermophor-associated glands as well as neurite bundles branching off the bulb nerve and projecting as far as the base of the embolus, corroborates the findings of previous studies [26, 27].

Richter et al. [29] defined a sensory organ in the simplest case as “nothing more than a cluster of receptor cells”. Therefore, even the clusters of neuronal somata present in the palpal organ of all investigated taxa do not necessarily have to be interpreted as clusters of interneurons – possibly constituting a hitherto overlooked palpal ganglion – but could also represent clusters of receptor neurons. Candidates of sensory organs could be the deeply internalized sensillum-like structures at the base of the embolus of *Kukulcania hibernalis* similar to those described for *Philodromus cespitum* [27]. Considering that putative sensory organs are present in the palpal organs of early branched-off araneomorph taxa (*Kukulcania*) as well as in more distal taxa (*Philodromus*), we assume that not only the presence of neurite bundles, but also sensory structures in the male palpal organ are widespread in spiders. Our data reveal that the palpal nerve is always made of several, distinctive neurite bundles, which can be seen as a split into multiple compartments separated from each other by complex glial sheath. In the insect peripheral nervous system, such nervous compartments are functionally differentiated into sensory or motor neurons [30]. Similarly, Foelix [31] describes combined afferent and efferent neurite bundles as the general pattern for peripheral nerves in the legs of arachnids. It therefore seems likely that the palpal nerve consists of afferents from sensillar receptor neurons as well as efferents projected from the brain over the subesophageal ganglion into the pedipalp. Both sensory and motor functions can play various roles during mating. For example, proprio- or chemoreceptive information received via the embolus during mating can provide information with respect to stresses and strains in the intromittend organ as already suggested for *Hickmania troglodytes* [26]. Sensory feedback received by the palpal organ can further be advantageous if it helps the male to adjust his investment during the mating process, depending on whether the female mated previously or not. Theoretically, a sensory structure might also help to trigger manipulation of rival sperm stored in the female sperm storage organs. Our findings can also help to understand common behaviours, like the “stroking” behaviour [32] or palpal movements during copulation [15]. The latter were shown to result in removal of a predecessor’s sperm from the female’s sperm storage site [33]. These questions bear upon our understanding of sexual selection in spiders. Detailed analyses of the spider copulatory organs in the light of our findings should be addressed in the future.

Previous studies hypothesized that the efferent fibres included in the bulb nerve directly innervate the glands present in the palpal organ [26, 27]. Our findings of neurite bundles near glandular tissue support this assumption. The exact function of the palpal glands, however, is unclear but it is assumed that they play a pivotal role in the uptake and release of sperm - one of the major puzzles in spider reproductive biology [34], reviewed in [35]. As the spermophor lumen is usually not empty even before sperm uptake, it is likely that associated glands discharge secretions into the lumen [15; Günter, Michalik and Uhl unpublished]. Consequently, sperm uptake might be realized by resorption of these secretions by the glandular epithelium. A glandular system also permits the reverse assumption that sperm extrusion might be carried out by expelling the seminal fluid from the spermophor during mating. Lamoral [13] already considered that sperm are expelled by glandular activity and assumed a neurohormonal process. A neurohormonal system, however, cannot explain the very short mating events that occur in many spider species [17], nor the very fast extrusion of mating plug material that is often produced in the bulb [27, 36]. Sperm extrusion might also be related to haemolymph pressure [15]. It was hypothesized, that local variations of haemolymph pressure inside the palpal organ might also play a role, especially because not all spider taxa possess a partly porous spermophor into which glandular secretions can be discharged [37, 38]. For example, in mesothelid spiders the spermophor is unsclerotized [39] and could therefore be compressed under increased external haemolymph pressure. Our study cannot solve these mysteries, particularly the release mechanism of sperm from the spermophor, but the presence of the bulb nerve and neuronal clusters near the glandular epithelium in all investigated taxa now provides a basis for further detailed analysis of the underlying processes.

## Conclusions

We found nervous tissue in the palpal organs of all investigated spider taxa, namely the (1) bulb nerve, which is a distal branch of the palpal nerve, (2) afferent or efferent neurite bundles projecting from the bulb nerve into various parts of the palpal organ, and (3) 1 or 2 cluster(s) of neuronal somata. Therefore, palpal innervation is part of the ground pattern of the order Araneae. Moreover, the presence of sensory organs in the palpal organs of various taxa strongly suggests that palpal organs are sensitive structures. Our findings open up new avenues for studies on spider reproduction, as sensitive palpal organs expand the sensory capacity of male spiders during mating beyond what was considered possible.

## Methods

### Specimen collection

Specimens were collected in areas around Greifswald, Germany (*Marpissa muscosa*, *Tetragnatha* sp.); at the University of Bialystok field station, Gugny, Poland, (*Larinia jeskovi*); in Club Cala Llenya, Ibiza, Spain (*Loxosceles rufescens*); Del Norte County, California, USA (*Hypochilus pococki*); in Buenos Aires, Argentina (*Kukulcania hibernalis*); taken from lab-reared populations (*Stegodyphus dumicola*) or purchased from a commercial breeder (*Davus fasciatus, Liphistius* sp.). For voucher information see Additional file 1.

### Fixation and embedding

All samples were processed for ultrastructural analysis. Primary fixation and dissection were carried out in ice-cooled Karnovsky’s fixative [40], following incubation in a Pelco “BioWave Pro” laboratory microwave in combination with a Pelco “Steady Temp Pro Thermo Cube” solid state cooling unit (both Ted Pella, Inc., Redding, California, USA) (except of *H. pococki*, which was fixed in the field using Karnovsky’s fixative). The BioWave protocol was set to three microwave-pulses of 2 min each, operated at a power of 300 W. Every pulse was followed by a short break of 2 min to allow the samples to cool down. The maximum temperature of the sample chamber was set to not exceed 30 °C during the whole microwaving process. Afterwards, samples were stored in Karnovsky’s fixative in the fridge. For further processing, samples were washed with sodium phosphate buffer for 2 × 15 min, followed by post fixation in a 2% osmium tetroxide solution (in deionized water) for 150 min in an opaque box at room temperature. Subsequently, the samples were washed with deionized water for 3 × 10 min, followed by dehydration using graded series of ethanol for 2 × 15 min per step. Embedding was carried out using the “EMbed812” resin embedding kit (Science Services GmbH, München, Germany). We used different mixtures of propylene oxide:resin as following: 2:1 (4 h) 1:1 (overnight), 1:2 (12 h) and 0:1 (2 h) for embedding. For pre-embedding (up to 1:2), we used resin that had previously been stored in the freezer. During the 0:1 step, the samples were transferred to a “VacuTherm” vacuum heating cabinet (Thermo Fisher Scientific, Waltham, Massachusetts, USA) and incubated at 40 °C and 100 mbar for 3 × 30 min. Between each step, the vacuum was released slowly and uprising air was removed. Polymerization of the resin blocks was carried out in a heating cabinet at 60 °C for a minimum of 24 h.

### Micro-computed tomography

For obtaining micro-CT data, all samples were scanned in cured blocks to ensure accordance with the data from histological sectioning. All scans were performed using a Zeiss Xradia XCT-200 (Carl Zeiss X-ray Microscopy, Inc., Pleasanton, California, USA) at different magnifications and source voltages according to the specific sample that was scanned.

### Semi-thin serial sectioning and digitalization

All blocks were prepared for serial semi-thin sectioning. Sectioning was carried out with a Leica EM UC6 ultramicrotome (Leica Microsystems GmbH, Wetzlar, Germany), using a DiATOME “histo Jumbo” diamond knife (Diatome Ltd., Nidau, Switzerland) at section-thicknesses of either 700 nm or 1000 nm, depending on the object size.

### Re-sectioning and transmission Electron microscopy

Ultra-thin sections for TEM analysis were obtained from selected semi-thin sections [see [41]] using a Diatome Ultra diamond knife. Selected ultra-thin sections were transferred onto formvar-coated copper slot grids (G2500C, Plano GmbH, Wetzlar, Germany), followed by staining with uranyl acetate and lead citrate for 4 min each. Sections were then examined under a JEOL JEM-1011 Transmission Electron Microscope operated at 80 kV. Images were taken with an Olympus “Mega View III” digital camera (Olympus K.K., Tokio, Japan) using an iTEM software package (iTEM Software, Whiteley, UK).

### Digital processing, co-registration and reconstruction

Semi-thin serial sections were digitalized using a customized Visionary Digital BK Plus imaging system (Dun, Inc., Palmyra, Virginia, USA). The processes of alignment, reconstruction and co-registration were carried out in Amira 6.4 (FEI Software, now Thermo Fisher Scientific, Waltham, Massachusetts, USA), mostly in accordance with Ruthensteiner [42] and Handschuh et al. [41].

### Graphical processing and further imaging

All overview images of the external palp morphology were taken using the Visionary Digital Imaging system. All image adjustments were carried out using either Adobe Photoshop CS6 (Adobe systems, Inc., San José, California, USA) or CorelDRAW 2017, Corel PHOTO-PAINT 2017 and Corel PaintShop Pro 2018 (all Corel Corp., Ottawa, Ontario, Canada).

The terminology for the description of the nervous tissue is based on the neuroanatomical glossary by Richter et al. [29]. Spider-specific terminology is based on the Spider Anatomy Ontology (SPD) [43].

## Supplementary information

**Additional file 1:** Appendix – Voucher Data (DOCX 15 kb)

## Data Availability

The datasets supporting the conclusions of this article are available from the authors upon request.
