# Supplementary material for: The innervation of the male copulatory organ of spiders (Araneae) – a comparative analysis
Source: Front Zool. 2019 Oct 24;16:39. doi: 10.1186/s12983-019-0337-6 (PMC6813115; doi:10.1186/s12983-019-0337-6)
Supplement: Supplementary file 1 — Additional file 1: Appendix – Voucher Data (DOCX 15 kb) [file 12983_2019_337_MOESM1_ESM.docx]

**Appendix – Voucher Data**

Liphistiidae: *Liphistius* sp., purchased from commercial breeder, det. M. Pechmann, ZIMG II/28664

Theraphosidae: *Davus fasciatus*, purchased from commercial breeder, det. T. M. Dederichs, ZIMG II/28665

Hypochilidae: *Hypochilus pococki*, Del Norte County, California, USA, June 2017, leg. P. Michalik & M. Ramirez, det. P. Michalik, ZIMG II/28666

Filistatidae: *Kukulcania hibernalis*, Buenos Aires, Argentina, December 2017, leg. M. Ramirez, det. P. Michalik, ZIMG II/28667

Sicariidae: *Loxosceles rufescens*, Club Cala Llenya, Ibiza, Spain, June 2018, leg. C.H.G. Müller, det. C.H.G. Müller, ZIMG II/28668

Eresidae: *Stegodyphus dumicola*, Lab-reared population, August 2018, leg. A. Junghanns, det. A. Junghanns, ZIMG II/28669

Araneidae: *Larinia jeskovi*, University of Białystok, Field station Gugny, Poland, August 2016, leg. P. Mouginot, det. P. Mouginot, ZIMG II/28670

Tetragnathidae: *Tetragnatha extensa*, Greifswald, M-V, Germany, August 2018, leg. P. Michalik, det. T.M. Dederichs, ZIMG II/28671

Tetragnathidae: *Tetragnatha montana*, Greifswald, M-V, Germany, August 2018, leg. P. Michalik, det. T.M. Dederichs, ZIMG II/28672

Salticidae: *Marpissa muscosa*, Greifswald, M-V, Germany, August 2018, leg. T.M. Dederichs, det. T.M. Dederichs, ZIMG II/28673
